# The application of implementation science methods in correctional health intervention research: a systematic review

**DOI:** 10.1186/s43058-023-00521-4

**Published:** 2023-11-24

**Authors:** Tonya B. Van Deinse, Melissa J. Zielinski, Stephanie Brooks Holliday, Brittany N. Rudd, Erika L. Crable

**Affiliations:** 1 University of North Carolina at Chapel Hill School of Social Work, Chapel Hill, USA; 2 University of Arkansas for Medical Sciences Psychiatric Research Institute, Little Rock, USA; 3 RAND Corporation, Santa Monica, USA; 4 University of Illinois at Chicago, Chicago, USA; 5 Department of Psychiatry, University of California San Diego, San Diego, USA

**Keywords:** Implementation science, Corrections, Prison, Probation, Jail, Health care, Health equity

## Abstract

**Background:**

Improving access to high-quality healthcare for individuals in correctional settings is critical to advancing health equity in the United States. Compared to the general population, criminal-legal involved individuals experience higher rates of chronic health conditions and poorer health outcomes. Implementation science frameworks and strategies offer useful tools to integrate health interventions into criminal-legal settings and to improve care. A review of implementation science in criminal-legal settings to date is necessary to advance future applications. This systematic review summarizes research that has harnessed implementation science to promote the uptake of effective health interventions in adult criminal-legal settings.

**Methods:**

A systematic review of seven databases (Academic Search Premier, Cumulative Index to Nursing and Allied Health Literature, PsycINFO, Social Work Abstracts, ProQuest Criminal Justice Database, ProQuest Sociological Abstracts, MEDLINE/PubMed) was conducted. Eligible studies used an implementation science framework to assess implementation outcomes, determinants, and/or implementation strategies in adult criminal-legal settings. Qualitative synthesis was used to extract and summarize settings, study designs, sample characteristics, methods, and application of implementation science methods. Implementation strategies were further analyzed using the Pragmatic Implementation Reporting Tool.

**Results:**

Twenty-four studies met inclusion criteria. Studies implemented interventions to address infectious diseases (*n*=9), substance use (*n*=6), mental health (*n*=5), co-occurring substance use and mental health (*n*=2), or other health conditions (*n*=2). Studies varied in their operationalization and description of guiding implementation frameworks/taxonomies. Sixteen studies reported implementation determinants and 12 studies measured implementation outcomes, with acceptability (*n*=5), feasibility (*n*=3), and reach (*n*=2) commonly assessed. Six studies tested implementation strategies. Systematic review results were used to generate recommendations for improving implementation success in criminal-legal contexts.

**Conclusions:**

The focus on implementation determinants in correctional health studies reflects the need to tailor implementation efforts to complex organizational and inter-agency contexts. Future studies should investigate policy factors that influence implementation success, design, and test implementation strategies tailored to determinants, and investigate a wider array of implementation outcomes relevant to criminal-legal settings, health interventions relevant to adult and juvenile populations, and health equity outcomes.

**Trial registration:**

A study protocol (CRD42020114111) was registered with Prospero.

**Supplementary Information:**

The online version contains supplementary material available at 10.1186/s43058-023-00521-4.

Contributions to the literature

- Implementation science approaches and strategies can promote the uptake of evidence-based healthcare in carceral settings and reduce health disparities for populations involved in the criminal-legal system, but future implementation efforts in these settings should build on prior research to optimize strategy design and outcomes.
- This systematic review summarizes the implementation science research on adult correctional health interventions to date to describe key implementation determinants, use of implementation strategies, and implementation outcomes.
- This review identified 24 studies, most of which focused on implementation determinants. Across these studies, factors related to the inner setting (e.g., jail, prison, community corrections agency) were most salient highlighting the complexity of implementing health interventions within correctional health settings.
- Study findings inform a number of future directions for integrating implementation science into correctional health intervention research, including focusing on multi-level factors that impact implementation of evidence-based practices, measuring implementation outcomes, and developing and specifying implementation strategies.

Strategies that increase access to evidence-based health interventions and practices in criminal-legal settings are needed to advance health equity outcomes in the United States (U.S.). Multi-level sociopolitical and structural factors have disproportionately elevated incarceration rates and time spent in custody for individuals from lower-income communities and communities of color. For example, minimum federal prison sentencing laws, “stop and frisk” policing practices and searches are associated with higher rates of criminal-legal involvement for individuals from lower-income, Black, and Latino communities compared to higher-income, White communities (Anti-drug Abuse Act of 1986, 100 Stat. 3207; [1, 2]). Incarceration and criminal-legal involvement are increasingly identified as impactful social determinants of health, given the negative consequences of these experiences on the health of individuals, their families, and communities during and after incarceration [3]. For example, compared to the general population, incarcerated individuals have a greater risk for contracting HIV and for developing many chronic health conditions including substance use disorders, mental illnesses, hypertension, and respiratory conditions [4–6]. Disproportionate rates of incarceration among communities of color, coupled with negative health correlates of incarceration, means that enhancing access to evidence-based healthcare in carceral settings is critical to reducing health disparities individuals experience during and after incarceration.

However, widespread access to evidence-based healthcare interventions in criminal-legal settings is poor [7, 8]. There is a need for knowledge about *how* to effectively bring best practices to these contexts. Implementation science, a field that aims to advance population health by accelerating the translation of evidence-based practices (EBPs) into routine practice under real-world conditions, is ideally suited to building the knowledge to advance this goal. However, there is little research on the application of implementation science in carceral and community supervision contexts, including the design and utility of implementation strategies needed to improve access to evidence-based healthcare for criminal-legal involved individuals—a gap which we address in this review.

**Implementation science**

Common implementation science study outcomes include measuring the acceptability, adoption, appropriateness, costs, feasibility, fidelity, and sustainability of a focal EBP [9]. Implementation science studies often examine determinants (e.g., barriers or facilitators) that impact the use of EBPs or promising practices. Often, these studies are guided by an implementation science framework describing the implementation process and various factors that influence implementation outcomes [10]. The Consolidated Framework for Implementation Research (CFIR; [11]) and the Exploration, Preparation, Implementation, Sustainment framework [12] are two of the most widely used implementation science frameworks [13, 14].

Implementation science studies also assess the impact of implementation strategies, which are the specific methods or actions (e.g., providing training or consultation, changing workflows) used to adopt and sustain the EBP [15]. Some implementation studies directly compare individual implementation strategies or implementation strategy packages to assess differential effectiveness. Implementation strategies should be designed to respond to identified determinants and other contextual factors including pre-conditions and moderators that can increase or decrease implementation success [16, 17]. Careful implementation strategy specification is critical to activating necessary processes or events (i.e., a mechanism) that ultimately makes the strategy useful in the setting where it is deployed.

To illustrate, consider a research team that is collaborating with a county jail to implement an EBP group treatment that aims to improve symptoms of serious mental illness during incarceration. Based on results of their previous work and other published studies, the research team knows that certain organizational factors (i.e., implementation determinants or contextual factors), such as lack of leadership engagement and lack of a referral protocol, have inhibited successful implementation of this EBP. Consequently, the research team decides to create an implementation strategy that responds to these contextual factors to enhance the EBP’s implementation. Specifically, the research team plans to utilize the jail’s existing database to develop an automatic prompt for referral that is based on the individual’s score on a routine screening for mental health needs which typically occurs within the first 24–48 hours after booking. A well-respected nurse who has been working at the jail for more than a decade (i.e., an implementation champion) will describe the new referral prompt and train health and corrections staff on how to access the prompt and refer individuals to the EBP. In this example, the database and the ability to create a prompt or flag is a precondition because the causal pathway between the implementation strategy (i.e., creating the referral prompt) is dependent upon a database that can create the prompt. The engagement of the nurse, who is a trusted leader or champion, is a moderator because the degree of engagement from this trusted leader can amplify or facilitate the uptake of the referral prompt process.

Given the myriad contextual factors impacting the successful implementation of health interventions within criminal-legal settings, implementation science offers a pragmatic set of frameworks and methods to identify, mitigate, or leverage key determinants, and test contextually specified implementation strategies to their ability to achieve EBP implementation success and improve health equity [18].

**Implementation science and criminal-legal settings**

To date, the National Institute on Drug Abuse (NIDA) has launched two initiatives focused on incorporating implementation science and strategies in adult criminal-legal settings: the Criminal Justice Drug Abuse Treatment Studies (CJ-DATS) and Justice Community Opioid Innovation Network (JCOIN). The first phase of CJ-DATS (i.e., CJ-DATS 1) was launched in 2002 [19] and was followed by a second phase (i.e., CJ-DATS 2) announced in 2007 [20]. The CJ-DATS studies aimed to improve public health and safety outcomes for individuals leaving incarceration by improving substance use treatment access in criminal-legal settings. CJ-DATS studies included efforts to implement HIV and hepatitis C services, medications for substance use disorders, and other behavioral healthcare. JCOIN launched in 2019 to test implementation strategies to adopt medications for opioid use disorder in criminal-legal settings and to engage and retain criminal-legal involved populations in opioid use treatment services. Although adolescents are not a focus of this study, it is important to note that NIDA also funded the Juvenile Justice – Translational Research on Interventions for Adolescents in the Legal System (JJ-TRIALS) initiative. JJ-TRIALS research was conducted between 2013 and 2018 across 33 sites with the goal of identifying effective implementation strategies for substance use and HIV prevention and treatment for criminal-legal involved youth [21]. Adult and youth corrections settings in the U.S. differ in their physical environments, programming, and lengths of incarceration are often much longer for adults than youths. The health needs and interventions often delivered to adults and youths also differ. Given these dissimilarities, this systematic review is focused on implementation research conducted with criminal-legal involved adults who make up the largest corrections population in the U.S.

Although NIDA’s initiatives are critical to advancing substance use treatment for criminal-legal involved populations, the number of studies funded by these initiatives relative to the need for research on EBP delivery in these settings was small (i.e., 32 projects at the time of publication) and the foci were constrained to the aforementioned topics. Moreover, the field of implementation science has grown exponentially during the interval between these two initiatives, and it is likely that researchers outside of these specific initiatives have applied implementation science approaches to facilitate health interventions in criminal-legal settings. There remains a need to (1) understand how implementation science frameworks, strategies, and approaches have been applied to support EBP use in criminal-legal settings to date so we can build on successful implementation strategies and prevent replication of unsuccessful approaches, and (2) to describe the full range of health conditions targeted and types of interventions implemented using implementation science approaches to address health inequities for criminal-legal involved individuals.

**The current review**

The goal of this systematic review is to summarize how researchers have harnessed implementation science approaches, including assessing determinants and developing and testing implementation strategies, to promote the uptake of effective health interventions, practices, and programs in carceral or community supervised settings. Based on these findings, we present recommendations to advance implementation science in criminal-legal settings with the goal of achieving greater health equity for criminal-legal involved populations.

## Methods

The research team conducted a systematic review in accordance with PRISMA guidelines [22]. A study protocol (CRD42020114111) was registered with Prospero prior to article searching and data extraction. The research team conducted an initial search in December 2019 and a secondary search for new publications in September 2021 due to COVID-related delays.

### Database sources and search strategy

The research team searched peer-reviewed literature registered in seven computerized article databases, including Academic Search Premier, Cumulative Index to Nursing and Allied Health Literature, PsycINFO, Social Work Abstracts, ProQuest Criminal Justice Database, ProQuest Sociological Abstracts, and MEDLINE/PubMed. The team also hand searched for relevant articles across four journals (i.e., the Journal of Offender Rehabilitation, Health and Justice, Implementation Science, and the Administration and Policy in Mental Health and Mental Health Services Research) to ensure that no articles were overlooked by database search criteria. Members of the research team met with a reference librarian to confirm search terms for three key concepts: implementation, corrections, and health intervention. A complete list of search terms is in Appendix 1.

### Inclusion criteria

Articles were included in the systematic review if they (1) described an empirical study of implementation outcomes, determinants, and/or implementation strategies related to a health intervention; (2) cited an implementation science theory, model, framework, or taxonomy in their approach; (3) were conducted within a criminal-legal setting (i.e., jails, prisons, community supervision, and courts); (4) focused on interventions for adult populations; (5) were conducted within the U.S.; (6) were published in a peer-reviewed journal, (7) were published in English, and (8) were published between January 1, 1998, and August 31, 2021. Since the implementation science field is relatively young, and most public health intervention research in criminal-legal settings began to increase during the 2000s, we selected 1998 as the starting year to capture implementation research. Studies were excluded if they solely described efficacy or effectiveness results of health interventions or EBPs (i.e., did not assess implementation outcomes), described a study protocol, viewpoint (e.g., conceptual articles, perspectives), systematic reviews, dissertations, conference abstracts, studies that focused on youth or juvenile justice populations (whose needs and correctional environments differ from those of the adult population), or were published outside of the aforementioned date range. Studies that were conducted outside of the U.S. were also excluded given the significant differences in criminal-legal and health service systems across the global community.

### Study screening, data abstraction and synthesis

Search results were managed using Zotero and uploaded into Covidence [23] for de-duplication, screening, and full text review. All abstracts were reviewed by two members of the research team. Disagreements over study eligibility were discussed until a consensus decision was reached or adjudicated by a third reviewer, and reasons for exclusion were documented. All articles eligible for full text review were evaluated by two reviewers, with consensus discussions and a third reviewer resolving any disagreements on article eligibility for the systematic review.

The team developed a standardized abstraction template to summarize study information including location, study aims, type of criminal-legal setting, description of the intervention being implemented, study design, sample characteristics, analytic approach, outcomes measured, implementation framework(s) applied, relevant framework domains named in the findings, contextual factors, a description of implementation strategies used and their corresponding targets. All extracted data were reviewed by the research team to ensure accuracy. Specific implementation outcomes (e.g., acceptability, adoption) identified in the studies were extracted and coded using Proctor et al.’s [9] taxonomy of implementation outcomes. Proctor et al.’s taxonomy of implementation outcomes is widely used and offers a summary of outcome measurement language referenced in many implementation theories, models, and frameworks. Several studies in this review used Proctor et al.’s taxonomy to define their implementation outcomes of interest, and those specific outcomes were extracted for this review. When authors described implementation outcomes that were not framed using the Proctor taxonomy, two reviewers consensus coded the implementation outcome based on its description in the study and then categorized the outcome using the Proctor taxonomy.

Implementation determinants were extracted and categorized based on the five domains of the Consolidated Framework for Implementation Research (CFIR; [11]). The research team selected this framework as it is one of the most widely cited [13] and recognizable implementation frameworks and was developed by consolidating domains and definitions from multiple implementation frameworks. Additionally, many studies in this review used CFIR to code their implementation determinants. When authors did not use the CFIR domains in their analysis, two reviewers consensus coded the implementation determinant based on its description in the study and then categorized the determinant using the CFIR domains to promote standardized terminology across our synthesis.

We operationalized implementation strategies reported in all implementation trials using the Pragmatic Implementation Reporting Tool [24]. The research team selected this tool because it provides a standardized approach to specifying implementation strategy components used in clinical and implementation research. The Pragmatic Implementation Reporting Tool integrates implementation strategy reporting guidelines from Proctor et al.’s “Specify It” criteria and Presseau et al.’s [25] Action, Actor, Context, Target, Time framework. Implementation trials were defined as studies that evaluated the effectiveness of an implementation strategy on a specified implementation outcome (e.g., the effectiveness of a “facilitation” strategy on adoption of an EBP). Implementation trials that did not report implementation outcomes were not included in this step of the coding. Using the Pragmatic Implementation Reporting Tool [24] as the guide, one reviewer examined all relevant implementation trial manuscripts and identified relevant protocol papers to extract and document information relating to the strategy specification. A second co-author reviewed the data extraction to promote rigor.

## Results

**Fig. 1:**
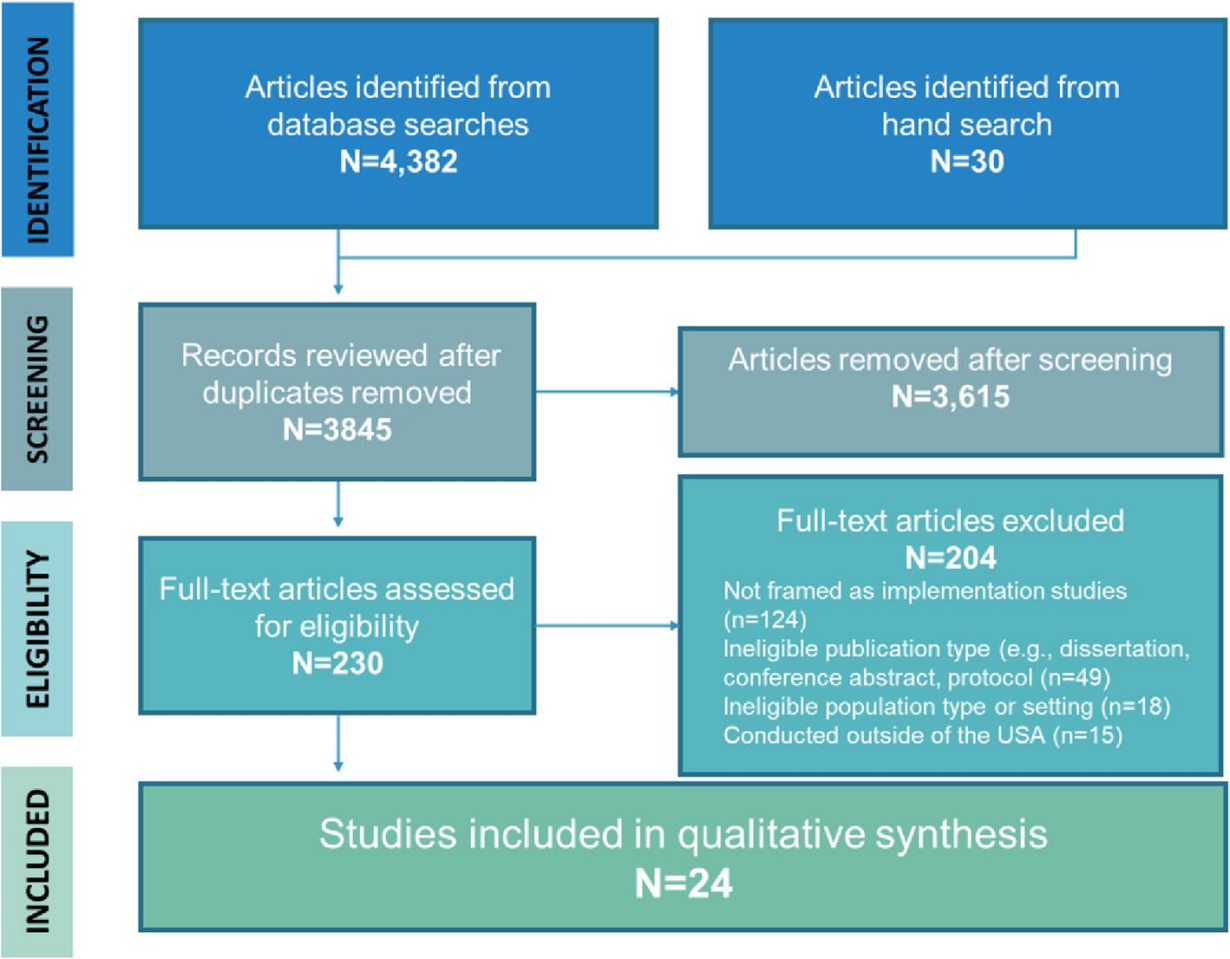
PRISMA flow chart

A total of 4382 articles were identified in the searched databases and 30 studies were identified through hand searching (Fig. 1). Of these, 230 articles met criteria for full text review. The majority of excluded articles did not frame the study as implementation science or cite implementation science relevant references (*n* = 124), were ineligible publication types (e.g., protocols, conference abstracts, *n* = 49), were not conducted in a criminal-legal setting (*n* = 18) or outside of the U.S. (*n* = 15). In total, 24 articles were included in the study sample (see Table 1).

**Table 1:** Study citation, purpose, methods

Citation	Purpose	Design, data, sample
[26]	Conduct a pre-implementation assessment to describe existing correctional HIV policies and practices and to identify gaps in HIV testing, prevention and treatment services.	Design: Observational: single point Data: Quantitative Sample: 9 state departments of correction, 2 county Sheriff's Departments, 23 prisons, 14 jails
[27]	Examine barriers and facilitators of the planning and implementation of a collaborative initiative between the Health Department and jail to provide HPV vaccinations	Design: Observational: single point Data: Qualitative Sample: 13 administrators and staff (7 from the health department and 6 from the jail)
[28]	Assess the implementation and sustainment of delivering medications for opioid use disorder in two jails and two prison systems.	Design: Observational: longitudinal Data: Qualitative, quantitative Sample: 4 study sites
[29]	Assess the effectiveness of an organizational linkage intervention on staff perceptions of and willingness to refer to addiction pharmacotherapy.	Design: Experimental (random assignment, experimental group) Data: Quantitative Sample: 20 sites (10 control, 10 experimental)
[30]	Conduct a formative evaluation of the implementation of a treatment intervention for justice-involved people with co-occurring mental health and substance use disorders.	Design: Observational: single point Data: Qualitative Sample: 28 meetings with stakeholders, 4 focus groups with staff, and policymakers, 2 focus groups with current participants , 10 interviews with program graduates
[31]	Assess the effectiveness and cost-effectiveness of interpersonal psychotherapy for women and men with major depressive disorder in a prison setting.	Design: Experimental (random assignment, experimental group) Data: Quantitative Sample: 181 people in prison
[32]	To describe perspectives on and implementation determinants of the implementation of interpersonal psychotherapy delivered a in prison settings.	Design: Observational: longitudinal Data: Qualitative, quantitative Sample: 71 providers and administrators from prison facilities; 90 people in prison
[33]	Compare the organizational readiness for change and other organizational factors related to evidence-based practice use across correctional settings and community-based residential and outpatient programs for substance use disorder.	Design: Observational: single point Data: Quantitative Sample: 165 correctional staff, 256 community residential, 267 community outpatient)
[34]	Examine organizational characteristics and processes that lead to successful implementation of HIV-STIC.	Design: Observational: single point Data: Qualitative Sample: 17 dyads of researchers and sponsors
[35]	Examine the influence of correctional agencies and community substance use treatment programs organizational structure as a determinant for providing HIV testing practices.	Design: Observational: single point Data: Quantitative Sample: 289 corrections administrators; 217 substance use treatment directors
[36]	Measure fidelity to the HIV Services and Treatment Implementation in Corrections (HIV-STIC) implementation strategy and document reasons for adapting and modifying the implementation strategy.	Design: Observational: longitudinal Data: Qualitative, quantitative Sample: 28 correctional facilities
[37]	Assess the impact of a process improvement intervention for improving HIV services for people under correctional supervision.	Design: Experimental (random assignment, experimental group) Data: Quantitative Sample: 14 research centers
[38]	Assess the feasibility of implementing the risk needs responsivity treatment planning support tool in a case management and peer support intervention for individuals with co-occurring disorders.	Design: Observational: single point Data: Qualitative, quantitative Sample: 55 participants in reentry program; 21 staff members
[39]	Identify organizational characteristics impacting the success of an organizational change strategy to improve assessment and case planning processes with people living with substance use disorder.	Design: Experimental (random assignment, experimental group) Data: Quantitative Sample: 659 staff members from 21 study sites
[40]	To describe the processes and strategies for translating correctional nursing standards into practice.	Design: Observational: longitudinal Data: Quantitative Sample: 444 correctional nurses
[41]	Explore implementation determinants of jail-based overdose education and naloxone distribution programming.	Design: Observational: single point Data: Qualitative Sample: 34 participants across 3 counties
[42]	To test the impact of a change team model implementation strategy on improving awareness of and linkage to HIV services and sustaining new services.	Design: Experimental (random assignment, experimental group) Data: Quantitative Sample: 2,301 people incarcerated
[43]	Assess determinants that impact implementation of specialty mental health probation.	Design: Observational: single point Data: Qualitative Sample: 26 staff members from probation and mental health and substance use services administrators
[44]	Describe implementation of specialty mental health probation, including perspectives on core intervention components, how probation officers approach implementation, and resources needed to optimize implementation.	Design: Observational: single point Data: Qualitative Sample: 16 probation officers and supervisors
[45]	Assess the impact of a change team model implementation strategy on perceived value of HIV services among staff of correctional and community HIV organizations.	Design: Experimental (random assignment, experimental group) Data: Quantitative Sample: 379 administrators and staff from 28 prisons, jails, or work release centers and community agencies
[46]	Assess the factors that impact sustainability of improvements made in a HIV service intervention.	Design: Experimental (random assignment, experimental group) Data: Qualitative Sample: 9 sites, including 9 principal investigators and 8 executive sponsors
[47]	Test an organizational linkage intervention using local change teams to connect individuals on community supervision to community-based medication-assisted treatment.	Design: Experimental (random assignment, experimental group) Data: Qualitative, quantitative Sample: 10 sites in the experimental group; 10 sites in TAU
[48]	To adapt and assess the effectiveness of an intervention to improve clinical and cultural competence for providing healthcare services to transgender clients.	Design: Pre-experimental (pre-test/post-test) Data: Qualitative, quantitative Sample: 34 correctional healthcare providers
[49]	Identify factors that contributed to implementation and sustainment of a therapy for survivors of sexual violence incarcerated in women's prisons.	Design: Observational: single point Data: Qualitative Sample: 13 program stakeholders, 22 incarcerated women

### Design, sample and setting characteristics of included studies

Table 2 summarizes the study design, sample, and setting-related characteristics of all studies included in this systematic review. In terms of study design, 42% (*n* = 10) of the articles identified were cross-sectional studies (i.e., data collected from a single measurement point), 28% (*n* = 9) were experimental (i.e., random assignment), and 21% (*n* = 5) were pre-experimental studies with pre- and post-test measures across a single group. Few (24%; *n* = 6) used a hybrid design, an increasingly common approach to addressing effectiveness and implementation aims within the same trial (see [50]). Approximately 42% (*n* = 10) exclusively employed quantitative methods, 33% (*n* = 8) used qualitative methods, and 25% (*n* = 6) used both qualitative and quantitative methods**.**

**Table 2:** Study characteristics and settings (*n* = 24)

Category	*N* (%)
Design	
Cross-sectional (i.e., single point), no comparison group	10 (41.7%)
Random assignment, treatment and control group (i.e., experimental)	9 (27.5%)
Pre-test/post-test, no comparison group (i.e., pre-experimental)	5 (20.8%)
Results drawn from hybrid design	6 (25.0%)
Data analysis	
Quantitative	10 (41.7%)
Qualitative	8 (33.3%)
Quantitative and qualitative	6 (25.0%)
Sample	
Criminal justice staff and community-based partners	8 (33.3%)
Administrative data	6 (25.0%)
Criminal justice staff	5 (20.8%)
Criminal justice staff and research partners	2 (8.3%)
Criminal justice staff, clients, community-based partners	2 (8.3%)
Community-based staff and clients	1 (4.2%)
Study setting(s)^1^	
Prison	17 (70.8%)
Jail	12 (50.0%)
Community corrections	7 (29.2%)
Work release	2 (8.3%)
Corrections setting not specified	1 (4.2%)
Primary health focus	
Infectious disease	9 (37.5%)
Substance use	6 (25.0%)
Mental health	5 (20.8%)
Co-occurring mental health and substance use	2 (8.3%)
Other	2 (8.3%)
Part of Criminal Justice Drug Abuse Treatment Studies (CJ-DATS)	11 (45.8%)

^1^Numbers will not add to 24 because 12 studies included multiple settings

Studies included representation from multiple criminal-legal staff, community-based partners, clients, and researchers. Few studies only included criminal-legal staff (21%, *n* = 5), while other studies included criminal-legal staff and community-based partners (33%, *n* = 8), or staff, community-based partners and clients (8% *n* = 2), or staff and research partners (8%, *n* = 2). One study included community-based staff and clients (4%), and 6 studies used administrative data (25%). Most studies (71%; *n* = 17) included at least one prison setting and half (*n* = 12) included at least one jail setting. Less than a third of studies (29%; *n* = 7) were conducted in a community corrections setting such as probation or parole. No identified studies focused on specialty courts (e.g., drug or mental health treatment courts).

Approximately 38% (*n* = 9) of studies investigated interventions to address infectious diseases, 25% (*n* = 6) addressed substance use, 21% (*n* =5) focused on mental health, and 8% (*n* = 2) addressed co-occurring mental health and substance use. Nearly half of all studies (46%, *n* = 11) were funded by either phase 1 or phase 2 of NIDA’s CJ-DATS initiative.

### Implementation focus and findings

Table 3 presents a summary of the implementation science approaches used in the design and/or analyses of the studies. Implementation science theories, models, frameworks, and taxonomies applied in these studies included the following: Proctor’s Implementation Research Model [51]; Consolidated Framework for Implementation Research [11]; Exploration, Preparation, Implementation, & Sustainment Framework [12]; TCU Program Change Model [52], Promoting Action on Research Implementation in Health Services [53]; Rogers’ Organizational Diffusion of Innovations [54, 55]; Proctor’s Taxonomy of Implementation Outcomes [9]; Proctor’s Implementation Strategy Specification [15]; Practical, Robust, Implementation and Sustainability Model [56]; Expert Recommendations for Implementing Change [57].

**Table 3:** Integration of Implementation Science Methods (*n*=24)

Category	*N* (%)
Implementation Focus	
Studies examining implementation determinants	16 (62.5%)
Studies examining implementation outcomes	12 (50.0%)
Studies evaluating implementation strategies	6 (25.0%)
Application of Implementation Science Frameworks and Taxonomies	
IS framework used for measure development and selection	12 (50.0%)
IS framework used for data analysis and coding	9 (37.5%)
IS framework used for framing introduction, study justification, discussion	16 (66.7%)
IS framework identified but not applied	2 (8.3%)
Unspecified/not enough info to code	1 (4.2%)
CFIR Domains Categorizing Implementation Determinants (*n* = 16)	
Inner setting	14 (87.5%)
Outer setting	10 (62.5%)
Implementation process	6 (37.5%)
Characteristics of individuals	5 (31.3%)
Intervention characteristics	5 (31.3%)
Implementation Outcomes (*n* = 12)	
Acceptability	5 (41.7%)
Feasibility	4 (33.3%)
Penetration/reach	2 (16.7%)
Appropriateness	1 (8.3%)
Fidelity	1 (8.3%)
Cost	1 (8.3%)
Sustainability	1 (8.3%)
Adoption	1 (8.3%)
Other	2 (16.7%)
Unspecified	1 (8.3%)

Of the 24 studies, 63% (*n* = 16) examined implementation determinants (e.g., barriers and facilitators). Examples of studies focused on implementation determinants include a pre-implementation assessment of factors that could impact intervention implementation (e.g., [26]), a formative evaluation of an intervention (e.g., [30]), a post-implementation assessment of provider perspectives on implementation of an intervention (e.g., [32, 43]), and a retrospective examination of factors impacting implementation and sustainment of an intervention (e.g., [49]). Of the 16 studies examining implementation determinants, factors impacting implementation were most commonly associated with the inner setting (i.e., the organization in which the intervention was implemented; 88%, *n* = 14), followed by the outer setting (63%, *n* = 10), implementation process (38%, *n* = 6), characteristics of individuals engaged in the implementation effort (31%, *n* = 5), and intervention characteristics (31%, *n* =5).

In addition, 50% (*n* = 12) of studies measured implementation outcomes. Studies examining implementation outcomes primarily cited those included in the Proctor et al. [9] taxonomy. Of the 12 studies examining implementation outcomes, 42% (*n* =5) measured acceptability, which is the perception that a service or practice is “agreeable, palatable, or satisfactory” ([9], p. 67). Further, 33% (*n* = 4) of studies measured feasibility or the extent to which an intervention can be used within an organization. In addition, 17% (*n* = 2) of studies measured penetration or reach which is the degree to which a practice was integrated within the service setting ([9], p. 70). Additional implementation outcomes measured were appropriateness (8%, *n* = 1), fidelity (8%, *n* = 1), cost (8%, *n* = 1), sustainability (8%, *n* = 1), and adoption (8%, *n* = 1).

Few studies explicitly tested implementation strategies (25%, *n* =6). Tested implementation strategies included the following: conducting a series of rapid cycle processes [46], conducting a local needs assessment and strategic planning process [37, 45, 46], creating a local change team comprised of cross-disciplinary and cross-agency staff [37, 45, 46], utilizing a training coach to support the improvement process [37, 45, 46], promoting network weaving through local councils [29, 47], and providing education and outreach (Friedman et al., 2015; [47, 48]). Three articles [37, 45, 46] examined the implementation strategies from the HIV Services and Treatment Implementation in Corrections (HIV-STIC) studies, which was part of the NIDA-funded CJ-DATS. Two studies [29, 47] examined the same organizational linkage strategy from the MAT Implementation in Community Correctional Environments (MATICCE) studies, also part of CJ-DATS. It is also important to note that the level of detail provided across studies was sometimes incomplete, and determinations related to the implementation strategy were based on the best available information within the included articles and their related protocols.

### Application of implementation science frameworks and taxonomies

Table 4 provides an overview of the implementation science methods used in each study. Although all studies cited a framework, application of the frameworks varied by study. Of the 24 studies included in this review, 50% (*n* = 10) used an implementation science framework or taxonomy to select or develop their data collection methods (e.g., [27, 33, 35]; Tables 1 and 4). For example, in some cases, the Proctor et al. [9] taxonomy was used to inform the researchers’ decisions about what implementation outcomes (e.g., acceptability, appropriateness) to select for a study and how to operationalize them (e.g., [45]). Additionally, implementation science frameworks were used for data analysis and coding for 38% (*n* = 9) of the studies (e.g., [30, 32, 43, 44, 49]). Most studies (67%; *n* = 16) applied frameworks in the introduction and discussion sections to frame the study and its results. In another 13% (*n* = 3) of the studies, an implementation science framework was identified or cited, but the authors did not specify how the framework was applied to their study or the authors did not include sufficient detail to code the application of the implementation science framework.

**Table 4:** Application of implementation science methods

Citation	Hybrid Design		Implementation focus			Application of implementation science framework			
	Not a hybrid	Hybrid Type 1 indicated	Assess determinants of EBP or strategy	Assess implementation outcomes	Test implementation strategy	IS framework identified but not applied	Measure development and selection	Data analysis and coding	Framed intro/discussion
[26]	x		x						x
[27]	x		x				x	x	
[28]	x		x	x				x	
[29]	x			x	x				x
[30]	x		x					x	
[31]		x		x		x			
[32]		x	x	x			x	x	x
[33]	x		x				x		x
[34]	x		x						x
[35]	x		x				x	x	x
[36]	x		x	x			x	x	x
[37]		x			x				x
[38]	x			x			x		x
[39]	x		x	x			x		x
[40]	x		x	x					x
[41]	x		x				x		x
[42]	x			x			x		
[43]		x	x					x	x
[44]		x	x				x	x	x
[45]	x			x	x		x		x
[46]		x	x	x	x				x
[47]	x				x	x			
[48]	x			x	x	x			
[49]	x		x				x	x	

## Discussion

This systematic review documented the use of implementation science theory and approaches in studies aiming to implement health interventions in criminal-legal settings. Our review identified 24 articles for inclusion that spanned a 14-year period from 2007 to 2021. Thus, on average, less than two articles each year were published that employed implementation science methods to researching the uptake of health interventions in criminal-legal settings. That correctional health research has not kept pace with advancements in implementation science research and methodology is troubling given the complex health needs of the 1.8 million people incarcerated in the nation’s prisons and jails and the 3.7 million people under community supervision [58–60]. Mass incarceration and complex co-occurring health conditions both disproportionately impact communities of color and low-income communities [61]. In addition to reforming the criminal-legal system to reduce these inequities, researchers can also investigate effective implementation strategies to rapidly increase access to high-quality health services that meet the health needs of people currently incarcerated and under community supervision. In this way, greater testing of dissemination and implementation strategies in criminal-legal settings can help address health inequities stemming from societal injustices of mass incarceration and the impact of criminal-legal involvement on social and economic wellbeing [62, 63]. This review provides a summary of published correctional health intervention research that integrated implementation science approaches and provides a foundation to inform future studies seeking to employ implementation frameworks, strategies, and outcomes in their study designs.

### Limitations

There are two notable limitations when considering the results of the study. First, it is possible that relevant studies may have been excluded within the parameters of the systematic search. Specifically, if a study was not framed as implementation science or clearly indicated the use of implementation science methods, it was excluded. For example, a study that examined barriers and facilitators of implementing a program but did not integrate implementation science within the study justification or methods would be excluded. This is also true for studies that may have been supported by funding from CJ-DATS but did not explicitly integrate implementation science methods. To include as many relevant studies as possible, we scanned articles for implementation science relevant citations that might indicate an implementation science approach. Second, the study used 1998 as a start date because of the initiation of Veterans Affairs Quality Enhancement Initiative (VA QUERI). Although most of the studies included in our review were from the 2010s, it is possible that earlier relevant studies could have been excluded. Finally, this systematic review focused on identifying implementation approaches, strategies, and outcomes from efforts aimed at providing evidence-based care to the adult criminal-legal involved population. Thus, we are unable to draw any lessons or comparisons from efforts conducted in juvenile justice settings (e.g., JJ-TRIALS). Adult and juvenile correctional settings differ in their populations, structure, and programming. Implementation strategies that are effective in juvenile settings where there is a stronger focus on healthcare and rehabilitation programming and support youths who typically serve shorter sentences, are likely to differ than implementation strategies used in adult correctional settings.

### Implications

Despite these limitations, this study also highlights a number of important future directions related to the implementation of EBPs in criminal-legal contexts.

#### Focus on determinants reflects complexity of the implementation environment

The fact that implementation determinants make up the majority of the studies in this review, most of which focus on the inner setting (i.e., the organizational context), reflects the need to understand the factors impacting implementation within a complex implementation environment. Correctional health interventions, by nature, often involve an inter-organizational and multi-disciplinary context in which practitioners who may be trained in one type of service (e.g., healthcare) are operating within the context of another agency environment (e.g., corrections). For example, a mental health intervention operated by a private service provider but co-located within a county-based detention center is cross-sectoral (i.e., private and public sector, respectively) and is susceptible to a broad range of multi-level factors originating from both the mental health service system and the criminal-legal system. Influences from both of these systems can create an implementation environment that may require significant intervention adaptation to render the intervention fit or appropriate for the implementation environment. Consequently, the fact that much of the implementation science focus in correctional health research has been about contextual inquiry (i.e., understanding implementation determinants) is appropriate and expected. Given the relative nascency of leveraging implementation science within correctional health research, and the variation in types of health interventions and correctional environments (i.e., jail, prison, courts, community supervision), the focus on contextual inquiry and implementation determinants should continue.

 Additionally, results show that much of the focus on implementation determinants has been on factors related to the inner setting, or the organization in which the intervention is being implemented. Although critically important, a singular focus on the organizational factors impacting intervention implementation does not account for significant external factors relevant in contextual inquiry. Notably, policy-level factors and those associated with inter-organizational relationships are significant determinants of whether and how an intervention is adopted into practice [64]. For example, recent opportunities to promote Medicaid enrollment prior to release from incarceration will impact costs and access to care in carceral settings and need to be considered in the implementation of new health interventions [65]. In addition, for interventions and implementation strategies focused on enhancing referral networks (e.g., [29, 47]) to increase service uptake, the presence, quality, and characteristics of inter-organizational relationships are critically important. Consequently, as researchers and practitioners continue to examine implementation determinants, a greater focus on domains beyond the inner setting is needed.

#### Increase the focus on implementation outcomes and strategies

Although contextual inquiry studies focused on implementation determinants are still needed, the field should also build on the knowledge produced by the two NIDA-funded initiatives, JCOIN and CJ-DATS. These initiatives accelerated the application of implementation science methods in correctional health interventions and focused on developing implementation strategies to enhance uptake of EBPs. Moving forward, researchers and practitioners can develop sequential study aims in which implementation determinants are studied in the first phase of research in a small pilot study and then addressed in subsequent stages, first through a pilot test of an implementation strategy and later through a larger study employing rigorous methods to test the efficacy of the implementation strategy. NIDA’s strategy of speeding up translation in correctional settings through federally funded consortium sites could be expanded to other institutes. Resulting studies from such initiatives would provide the field with invaluable information about the variation in the factors that impact implementation by fields of practice and health foci (e.g., substance use, mental health, infectious diseases) and the implementation strategies that enhance uptake of their respective EBPs. In addition to federal funding initiatives to speed up translation, researchers can consider using hybrid designs. Hybrid effective implementation designs challenge the typical sequencing of efficacy, effectiveness, and implementation research by promoting simultaneous examination of effectiveness and implementation aims [50, 66].

#### Standardize specification of implementation science methods

Assuming greater focus on the application of implementation science methods in correctional health research moving forward, it is imperative that researchers and practitioners standardize specification of methods in their reports and articles. Better specification will make it easier for other researchers and practitioners to understand, adapt, and apply these methods to their work and advance the research. Suggestions for better specification of implementation science methods in correctional health research articles include (1) providing a clear justification of the use of implementation science methods, including citations; (2) clear description of the implementation focus (i.e., implementation determinants, outcomes, and strategies) as well as the correctional setting (i.e., prison, jail, court, community supervision) and health focus (e.g., mental health, substance use, infectious disease); (3) identification, justification, and meaningful operationalization, and integration of selected implementation science frameworks throughout the study (e.g., include detailed descriptions of the chosen framework(s) and rationale; explain how the framework guided the study methods, such as instrument development, data collection, data analysis; describe the degree to which the framework fit the study context; explain how the framework can aid in the interpretation of results and transferability); (4) clear definition of implementation outcomes that map onto an implementation framework; and (5) standardized specification of implementation strategies, preferably using a framework (e.g., [15, 24]). Standardizing these methods helps to align the correctional health research with other health service fields to help better understand the role of the correctional context in implementation of EBPs.

## Conclusion

Although application of implementation science methods in correctional health intervention research is limited, integration of these methods appears to be accelerating, likely fueled by federally funded implementation-focused research consortiums. Overall, the implementation research on correctional health interventions has largely focused on understanding the environment in which health interventions are implemented. Although focusing on these implementation determinants is necessary given the complex environment in which health interventions are implemented, the field should increase its focus on developing implementation strategies to address the known factors that impede successful implementation and to standardize the way that implementation science methods are specified in correctional health research.

### Supplementary Information

**Additional file 1.**

## Data Availability

Data analyzed in this study may be made available from the corresponding author upon reasonable request.

## Abbreviations

CJ-DATS: Criminal Justice Drug Abuse Treatment Studies
CFIR: Consolidated Framework for Implementation Research
EBP: Evidence-based practices
EPIS: Exploration Preparation Implementation Sustainment
JCOIN: Justice Community Opioid Innovation Network
JJ-TRIALS: Juvenile Justice – Translational Research on Interventions for Adolescents in the Legal System
MATICCE: MAT Implementation in Community Correctional Environments
NIDA: National Institute of Drug Abuse
VA QUERI: Veterans Affairs Quality Enhancement Initiative
