# Supplementary material for: The application of implementation science methods in correctional health intervention research: a systematic review
Source: Implement Sci Commun. 2023 Nov 24;4:149. doi: 10.1186/s43058-023-00521-4 (PMC10675852; doi:10.1186/s43058-023-00521-4)
Supplement: Supplementary file 1 — Additional file 1. [file 43058_2023_521_MOESM1_ESM.docx]

Additional file

Appendix 1

Search Terms

| Terms | Search words |
| --- | --- |
| Implementation | implementation science, implementation research, dissemination, translational research, implementation study, implementation strategy, implementation determinants, implementation outcomes, contextual inquiry, strategy development, fidelity, diffusion, hybrid type, sustainability, adherence, acceptability |
| Corrections | Jail, prison, parole, probation, detention center, community corrections, court, mental health court , drug treatment court, incarceration, inmate, correctional facility, correctional supervision, community supervision, re-entry, post-release |
| Health intervention | Treatment, healthcare, intervention, screening process, program, assessment |
